# Reference Phase Stabilizer for Distributed Underwater Sonar Systems

**DOI:** 10.3390/s18124279

**Published:** 2018-12-05

**Authors:** Lijie Yang, Ruirui Dang, Chunyi Song, Zhiwei Xu

**Affiliations:** The Institute of Marine Electronic and Intelligent System, Ocean College, Zhejiang University, Zhoushan 316021, China; yanglijie@zju.edu.cn (L.Y.); dangruirui@zju.edu.cn (R.D.)

**Keywords:** distributed sonar systems, reference dissemination, phase synchronization

## Abstract

An optical fiber is a promising approach for data and clock transmission in distributed underwater sonar systems. However, synchronization is a critical challenge in distributed sonar systems, which mandates accurate clock synchronization down to a sub degree. Potential phase misalignment is caused by fiber length variations. In this paper, we propose a fiber-based phase stabilizer method to achieve accurate clock synchronization among sensor nodes. We use fiber-based feedback loop between sensor nodes and central station unit to monitor phase variations. Subsequently, we leverage phase shifters symmetrically arranged on the forward lane and feedback lane to compensate real-time phase variation and maintain high-precision synchronization. Besides, an ambiguity eliminator circuit is designed to remove the clock’s cyclic ambiguity. Both analysis and experimental results suggest that the proposed phase stabilizer can achieve 10 MHz reference clock synchronization within 0.4 degree. We also analyze the impact of the reference clock’s phase error on the system range detection accuracy, which indicates that the proposed phase stabilizer can greatly improve detection accuracy of sonar systems.

## 1. Introduction

A distributed sonar system utilizes a scalable number of collaborative sensors to detect and track targets over large underwater areas, and can achieve increased coverage and reliability compared to a traditional towed sonar system. The system can also improve the spatial resolution of detection, boost the accuracy of target parameter estimation and resilience to signal fading [1]. Detailed descriptions about distributed sonar system can be found in Refs. [2,3,4] and “Distributed Agile Submarine Hunting (DASH)” program disclosed by the Defense Advanced Research Projects Agency (DARPA) [5]. To accurately measure the direction of arrival (DOA) of echoed signals, all sensor nodes share a highly stable reference signal. This reference signal is provided by the central station and disseminated to all sensor nodes through optical fibers with different lengths. This could induce misaligned reference signals at different sensor nodes. Besides, different temperature and mechanical expansions of fibers could create fiber length variations. To stabilize the reference phase of each sensor node, these issues must be resolved.

Unlike terrestrial centralized radars that can precisely control the length of interconnection cables, underwater sonar systems do not have such flexibility and need other ways to guarantee synchronization among sensor nodes. In Refs. [6,7,8], several wireless synchronization algorithms have been proposed. However, these algorithms can not completely overcome the bad influence of long propagation delays caused by the low propagation speed of underwater acoustic communication. Refs. [9,10] use the global positioning system (GPS) to realize synchronization between sensor nodes, which is hard to achieve for underwater acoustic sonars. In Ref. [11], all sensor nodes in the network are synchronized by a same clock via coaxial cables. This is applicable to compact sensor networks where short range coaxial cable attenuation is negligible. However, distributed sensor nodes are scattered hundreds to thousands of meters away and are vulnerable to attenuation and electromagnetic interferences. Hence, this method may not be optimum.

Compared to coaxial cables, optic fiber links have broad bandwidth, low propagation loss, immunity to electronic magnetic interference and lighter weight [12,13,14,15,16,17,18]. Hiskett [19] proposes an optical communication system by utilizing 450 nm laser diode. The clock synchronization is realized by using reference headers appended to the encoded message signal, which the receiver node uses to correct for timing drift. However, complicating clock recovery strategies are required. Refs. [20,21,22,23,24] use controlled optical delays to compensate variation of the propagation delay of the fiber. However, optical devices, such as fiber stretchers, polarizing beam splitters, Faraday mirrors and optical frequency shifters, are bulky, and are also very challenging to integrate and increase manufacturing and maintenance cost. Therefore, an optical approach is not optimum for underwater applications. Alternatively, we can use an electrical approach based on compact electrical variable delay lines, which is easy to integrate and implement by a standard CMOS process. It has advantages in system size, power consumption and costs over optical solutions.

To achieve synchronization, the Square Kilometer Array [25] disseminates a high-stable reference signal to remote sites via optical fibers. An electrical phase–noise compensation circuitry is applied at the receiving site. Though it demonstrates good performance, the approach mandates large space with complicated compensation circuitries. Refs. [26,27] developed a dedicated integrated delay line circuit to compensate the fiber length variation. By exploiting bidirectional transmission in optical fiber links and placing two symmetrical delay lines at the transmitting site, it can achieve effective cancellation of the clock misalignment induced by fiber length variation. However, this method requires a highly stable reference frequency source like hydrogen maser, which (e.g., VCH-1005) is typically expensive, bulky and heavy.

In this paper, we introduce a reference phase stabilizing method for underwater distributed sonar systems. The proposed phase stabilizer is composed of a fiber-based phase lock loop (PLL) with cascaded phase shifters and an ambiguity eliminator circuit. The proposed phase stabilizer can achieve three advantages over prior methods. First, compared to phase stabilizer using optical devices, the proposed phase stabilizer uses electrical devices, which facilitates miniaturization and integration. Second, previous electrical phase stabilizers have never dealt with reference clock’s cyclic ambiguity, which cannot accurately compensate reference clock’s cyclic phase misalignment with long-distance optical transmission, and lead to reference asynchronization. The proposed phase stabilizer exploits an ambiguity eliminator circuit to successfully remove the reference clock’s cyclic ambiguity. Third, the proposed phase stabilizer offers a flexible phase adjustment range. The phase stabilizer proposed by Ref. [26] can also stabilize the reference phase, which, however, lacks flexibility. Its maximal adjustable range is 90 ns and constrains its applications. The proposed phase stabilizer designs a method to extend the adjustable range by simply cascading more phase shifters. Therefore, it expands its applications to different scenarios.

The remainder of this paper is organized as follows. Section 2 describes the principle of the phase stabilizer. Section 3 discloses the test bench we built to verify the proposed phase stabilizer. Section 4 concludes the paper.

## 2. Proposed Phase Stabilizer

**Figure 1:**
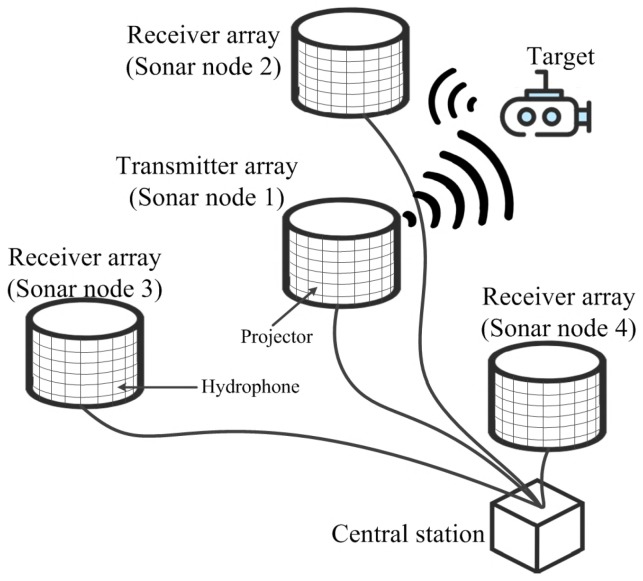
Structure of the adopted distributed sonar system.

The structural demonstration of the distributed sonar system adopted in this paper is shown in Figure 1, containing one central station and several remote sensor nodes. The sensor nodes are divided into one transmitter array and several receiver arrays. All sensor nodes are connected with the central station with fibers of different length, which is also called multistatic sonar system. The transmitter array emits pulses of sounds to target, and the receiver arrays acquire the echoes from the target. Also, some pulses of sounds are directly received by receiver arrays and are referred as direct path waves. Signal processors on the receiver arrays estimate the target azimuth angle and the distance between the target and the receiver arrays, which highly rely on the high-accuracy reference clock synchronization among sensor nodes to achieve desired DOA estimation.

**Figure 2:**
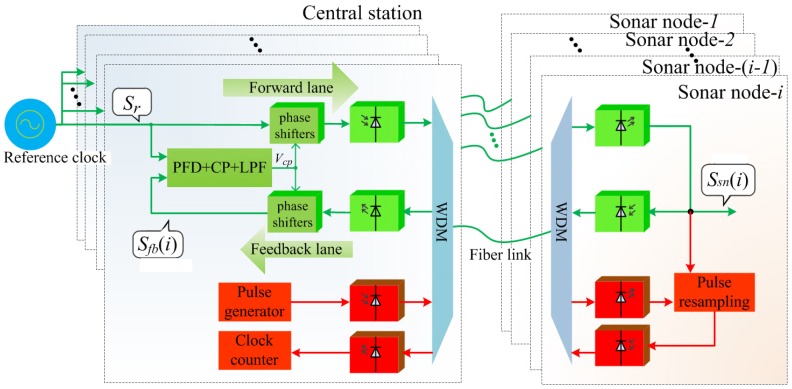
Block diagram of the proposed phase stabilizer for a distributed sonar system.

The reference phase misalignment among sensor nodes is due to the differences of optical fibers connecting sensor nodes. We propose an electronic phase stabilizer that can compensate fiber length variations. The proposed phase stabilizer is mainly composed of two parts: a fiber-based PLL with cascaded phase shifters (shown as green blocks in Figure 2) to eliminate fractional phase misalignment, and an ambiguity eliminator circuit (shown as red blocks in Figure 2) to eliminate cyclic ambiguity among reference clocks.

### 2.1. Fiber-Based PLL with Cascaded Phase Shifters

The fiber-based PLL is used to disseminate reference signals to each sensor node and measure the feedback signal’s phase constantly to compensate their mismatches due to fiber length variation. PLL is considered an effective approach to maintain signal phase, which is widely used for reference signal distribution and synchronization [28,29]. A conventional PLL comprises of a phase frequency detector (PFD) to detect the phase difference between forward signal and feedback signal, a charge pump (CP) and a low-pass loop filter (LPF) to generate output voltage $v_{cp}$ proportional to the phase difference, a VCO whose instantaneous oscillation frequency is determined by control voltage $v_{cp}$ [30]. We replace the VCO located at the forward lane with *n* cascaded phase shifters whose phase shift values are controlled by $v_{cp}$. Furthermore, we add *n* identical cascaded phase shifters at the feedback lane to ensure symmetry. The fiber-based PLL is illustrated as green blocks in Figure 2.

Plenty of remote sonar nodes are connected with the central station through different optical fibers. We take the *i*-th sensor node as an example. Assume the original reference signal generated on the central station board is

(1) $$S_{r}=cos\left(w_{r}t+\phi_{r}\right)$$

where $w_{r}$, $\phi_{r}$ are the frequency and initial phase of this original reference clock.

After passing through *n* cascaded phase shifters on the forward lane, the reference signal is converted into 1550 nm modulated light by an electric-photo transducer and wavelength division multiplexer (WDM). Then, the modulated reference signal is transmitted to the remote sensor node through optical fiber link. At the sensor node side, the 1550 nm modulated light is transformed back to electronic reference signal by another WDM and photo-electric transducer. However, the recovered reference signal’s phase becomes unknown due to the fiber length variation. We then transmit this reference signal back to the central station via the same fiber. The central station receives this feedback signal and delivers it to *n* cascaded phase shifters on the feedback lane. The control pins of phase shifters are connected together to the same tuning voltage, $v_{cp}$, guaranteeing identical phase shifts for both forward and feedback lanes. The output signal of these cascaded phase shifters on the feedback lane is delivered to the second input of the PFD to close the feedback loop.

The unidirectional phase drift caused by the fiber connecting the *i*-th sonar node and central station is defined as $\phi_{f}\left(i\right)$, which is given by

(2) $$\phi_{f}\left(i\right)=2\pi \cdot \frac{L(i)}{\lambda }$$

where λ is the wavelength of transmitted optical signal. L(i) is the length of fiber.

Other than optical fibers, components along the round-trip route also induce additional phase delays to the signal, such as electronic-optical transducers. In the proposed system, these components are distributed symmetrically on the forward lane and feedback lane. We then define these miscellaneous phase delays on either the forward lane or feedback lane as $\phi_{m}$. $\phi_{m}$ can be consistent among all sonar nodes by careful trimming during installation. However, $\phi_{f}\left(i\right)$ is derived from fiber length variation and can not be manually compensated.

If the phase shift introduced by every phase shifter is defined as $\phi_{ps}\left(i\right)$, the reference signal received by the *i*-th sensor node is expressed as

(3) $$S_{sn}\left(i\right)=cos\left[w_{r}t+\phi_{r}+n\phi_{ps}\left(i\right)+\phi_{f}\left(i\right)+\phi_{m}\right]$$

$S_{sn}\left(i\right)$ is a duplication of original reference signal, $S_{r}$, except for the phase delay derived from *n* cascaded phase shifters and fiber length variation. $S_{sn}\left(i\right)$ is then sent back to the central station through the same optic fiber. After passing through *n* phase shifters on the feedback lane, the feedback signal at the PFD’s second input is expressed as

(4) $$S_{fb}\left(i\right)=cos\left[w_{r}t+\phi_{r}+2n\phi_{ps}\left(i\right)+2\phi_{f}\left(i\right)+2\phi_{m}\right]$$

Similar to $S_{sn}\left(i\right)$, $S_{fb}\left(i\right)$ is also a delayed duplication of original reference signal, $S_{r}$.

In Figure 2, the PFD and CP on the central station board compare the phase difference between $S_{r}$ and $S_{fb}\left(i\right)$, and generate an error signal accordingly. The error signal passes through a LPF which removes high frequency elements of the error signal. Once through the filter, the error signal is applied to the control inputs of 2n phase shifters as their tuning voltage. Due to negative feedback nature, the tuning voltage controls phase shifters to reduce the phase difference between $S_{r}$ and $S_{fb}\left(i\right)$. Initially the loop is out of lock, and the error voltage pulls $S_{fb}\left(i\right)$ towards in-phase with $S_{r}$, until it cannot reduce the error any further and the loop is locked.

However, the PLL cannot distinguish a cycle difference between $S_{r}$ and $S_{fb}\left(i\right)$ and introduces cycle ambiguity. Practically, even when the loop is locked, the phase difference between $S_{r}$ and $S_{fb}\left(i\right)$ could be an integer number of reference cycles. That is

(5) $$2n\phi_{ps}\left(i\right)+2\phi_{f}\left(i\right)+2\phi_{m}=2k\pi ,k\in N$$

Substituting Equation (5) into Equation (3), we have

(6) $$S_{sn}\left(i\right)=cos\left(w_{r}t+\phi_{r}+k\pi \right),k\in N$$

Compared to Equation (3), Equation (6) reveals that the phase drift $\phi_{f}\left(i\right)$ due to fiber length variation can be successfully eliminated by the proposed PLL circuit. On the other hand, the proposed PLL introduces cyclic ambiguity to $S_{sn}\left(i\right)$. Assume a transmitter array and a receiver array are connected with the central station by fibers of 100 km and 60 km, respectively. The wavelength of a 10-MHz reference clock transmitted in the optic fiber is 20 m. Hence, the transmitter array and receiver array receive this reference clock with time delay of 5000 cycles and 3000 cycles, respectively. There is a 2000 cycle time difference that cannot be removed by the proposed PLL. To resolve this ambiguity, we proposed an ambiguity eliminator circuit in Section 2.2.

### 2.2. Ambiguity Eliminator

The ambiguity eliminator circuit is shown as red blocks in Figure 2, comprising of a pulse generator block, a pulse resampling block and a clock counter. The ambiguity eliminator circuit is used to measure the reference clock’s round-trip cyclic number, i.e., *k* in Equation (6), for every sensor node. With knowledge of *k*, the central station is able to schedule the dissemination time of reference clock to each sensor node and guarantee that all sensor nodes receive the reference clock at the same time, regardless of fiber length difference.

**Figure 3:**
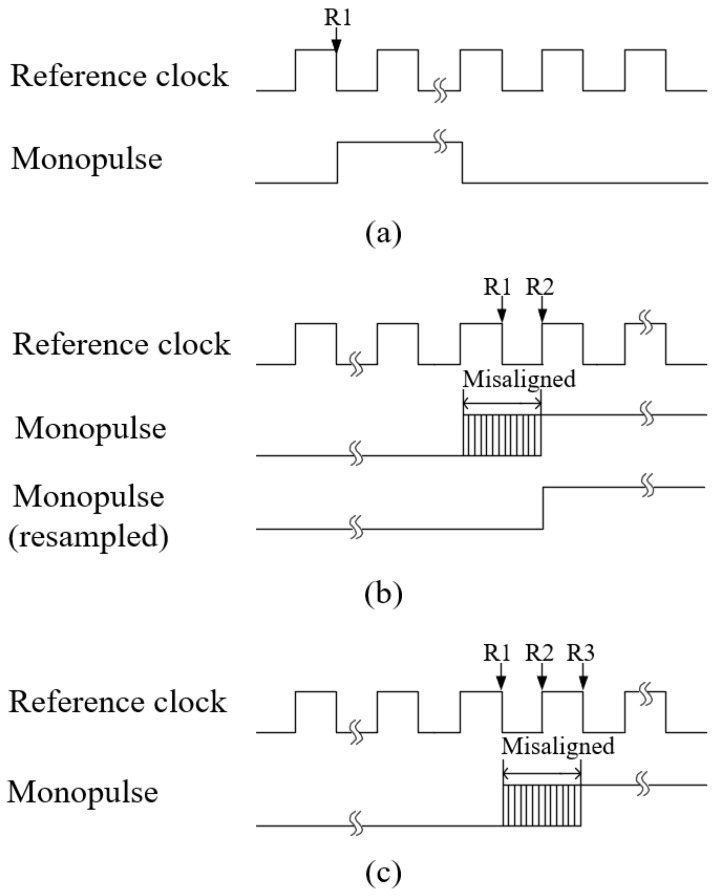
The timing of reference clock and monopulse. (**a**) Reference clock and monopulse generated at the central station. (**b**) Reference clock and monopulse received by the sonar node. (**c**) The loopback signals at the central station.

Taking the *i*-th sensor node as an example, the working mechanism of ambiguity eliminator circuit is as follows: The pulse generator generates a monopulse at the falling edge of the reference clock. Meanwhile, the clock counter starts to record the reference clock’s cyclic number. This monopulse is converted into an optical signal and sent to the sensor node along with the reference clock. The timing of reference clock and monopulse on the central station board is shown in Figure 3a, where the monopulse is asserted at the fall edge of reference clock, R1. At the sonar node, due to the phase shifters on the forward lane, the reference clock is slightly misaligned with the monopulse. The misalignment range is ±180°. To eliminate this misalignment, the pulse resampling block on the sonar node is used to sample this monopulse at the rising edge of the reference clock. The resampled monopulse is then aligned to R2, i.e., the next rising edge of R1. The timing on the sonar node is shown in Figure 3b. Then, this resampled monopulse is sent back to the central station. Similarly, the monopulse is misaligned with the feedback reference clock due to the phase shifters on the feedback lane and can be recovered at R3, i.e., the next falling edge of R1. Once the clock counter block detects the rising edge of recovered monopulse, it stops counting and obtains the reference clock’s cyclic number, *k*.

### 2.3. Three Operational States of the PLL

**Figure 4:**
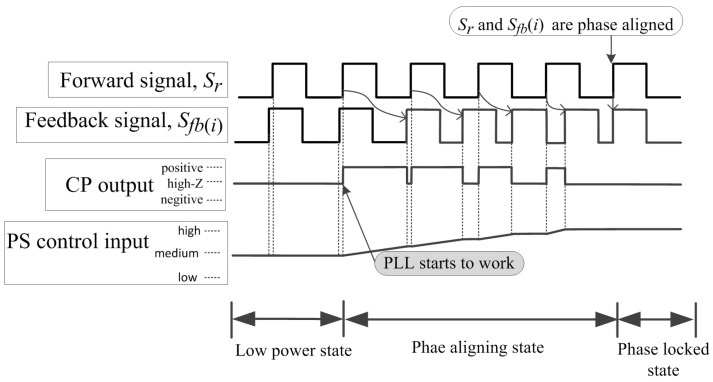
The changes of the CP’s output and phase shifter’s control input when $S_{r}$ is captured first. The phase shifter’s control voltage rises proportionally to the phase difference, until these two signals are phase aligned.

**Figure 5:**
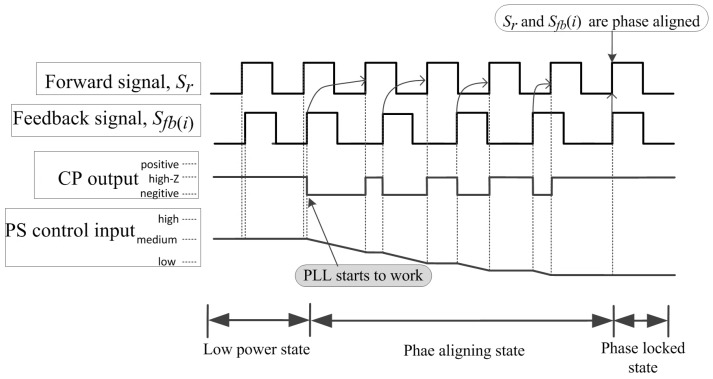
The changes of the CP’s output and phase shifter’s control input when $S_{fb}\left(i\right)$ is captured first.

The proposed PLL in the central station is the key module for the proposed phase stabilizer. The operational states of the PLL in the central station can be categorized into three states: low power state, phase aligning state and phase locked state. In low power state, the PFD ignores all input signals and the output of CP is in high-impedance mode. Hence, the phase shifters are unmodulated and the reference signals among sonar nodes are free-running. When the controller on the central station activates the PFD into a phase aligning state, the PFD starts to capture edges of $S_{r}$ and $S_{fb}\left(i\right)$. The CP is only active for a portion of each phase detector cycle that is proportional to the phase difference between $S_{r}$ and $S_{fb}\left(i\right)$. The output signal of the CP serves as a current sink or source, depending on which signal is captured first. The loop filter integrates this current and results in a continuously changing control voltage applied to the phase shifters. If $S_{r}$ is captured by the PFD first, the CP outputs a sequence of positive pulses, whose duty-cycle is proportional to the phase difference, as shown in Figure 4. Otherwise, if $S_{fb}\left(i\right)$ is captured first, the CP outputs a sequence of negative pulses, whose duty-cycle is proportional to the phase difference, as shown in Figure 5. Once the phase difference between the two signals reaches zero, the system enters the phase locked state, where the PFD’s output is narrow spurs. These current spurs are due to the finite speed of the logic circuits inside the PFD and have to be filtered by the loop filter so they do not modulate the phase shifters.

### 2.4. $v_{cp}$ Amplifying Circuit

**Figure 6:**
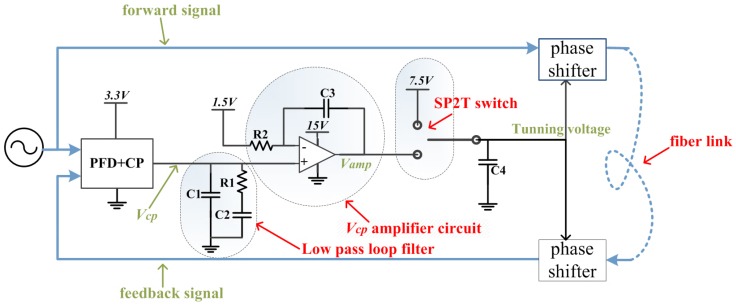
The detailed circuit description of the $v_{cp}$ process module, composed of a LPF, a $v_{cp}$ amplifying circuit and a SP2T switch.

Usually, the phase shifter’s control pin requires voltage much higher than the LPF’s output voltage. For instance, in this paper, the swing of LPF’s output voltage is only 0–3 V, while the phase shifter’s control input pin requires a 0–15 V tunning voltage. To match them, we design a $v_{cp}$ amplifying circuit to amplify LPF’s output voltage, $v_{cp}$, as shown in Figure 6. The circuit’s output $v_{amp}$ is expressed as

(7) $$v_{amp}\left(t\right)=\frac{1}{R_{2}C_{3}}\int \left[v_{cp}\left(t\right)-1.5\right]dt+v_{cp}\left(t\right)+v_{0}$$

Here $v_{0}$ is the initial state of $v_{amp}$.

The integrator in Equation (7) is key to determine $v_{amp}$. When the PLL is locked, $v_{amp}$ keeps constant. Any phase misalignment causes PLL to be unlocked and force $v_{cp}$ to go up or down until the PLL is locked again. Meanwhile, $v_{amp}$ changes with $v_{cp}$ until $v_{cp}$ returns to 1.5 V with the large integrator gain. We use a rail to rail amplifier here. The swing range of $v_{amp}$ is determined by the amplifier’s power supply (set to 15 V). The integration time is controlled by properly choosing $R_{2}$ and $C_{3}$.

The SP2T switch in Figure 6 is used to set the initial voltage of $v_{amp}$. The upper input pin of the switch is set to 7.5 V. The lower input pin is connected to the amplifier’s output. The switch’s output pin is directly connected to the phase shifters’ control pins. A shunt capacitor is connected at the switch’s output pin, which is used to avoid sudden voltage change at the phase shifters’ control pins when switch is shifted from the upper input to the lower input. Initially, the PLL is not working and the switch’s output pin is connected to the upper 7.5 V input, i.e., the median value of the full input range. When the phase stabilizer is activated, the lower input pin of the switch is connected to the phase shifters’ control pins. At the moment of switching event, the shunt capacitor $C_{4}$ at the output pin clamps $v_{amp}$ at 7.5 V to avoid sudden voltage change. Subsequently, the PFD, LPF and $v_{cp}$ amplifying circuit start to response to the phase difference of $S_{r}$ and $S_{fb}\left(i\right)$ until they are phase aligned.

## 3. Measurement

**Figure 7:**
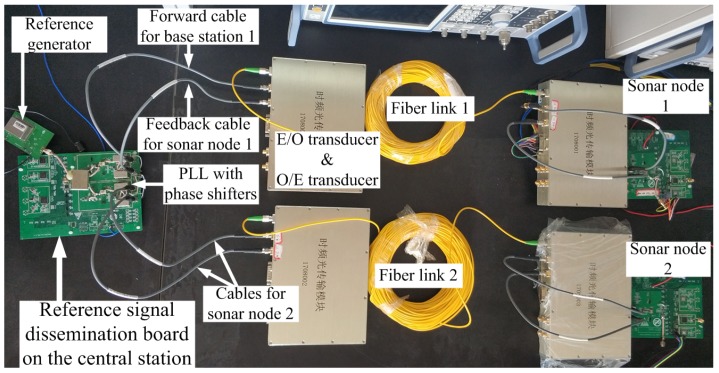
Top view of the proposed phase stabilizer evaluation system.

To validate the proposed phase stabilizer, we construct an evaluation system, as shown in Figure 7. The system contains one reference dissemination board to emulate a central station and two remote boards to emulate sensor nodes. Sensor node boards are connected with the reference dissemination board with optical fibers. The designed PLLs and phase shifters are at the right edge of the reference dissemination board. To verify the effectiveness of proposed phase stabilizer, we use fibers with different lengths, namely, 60 m and 100 m.

**Figure 8:**
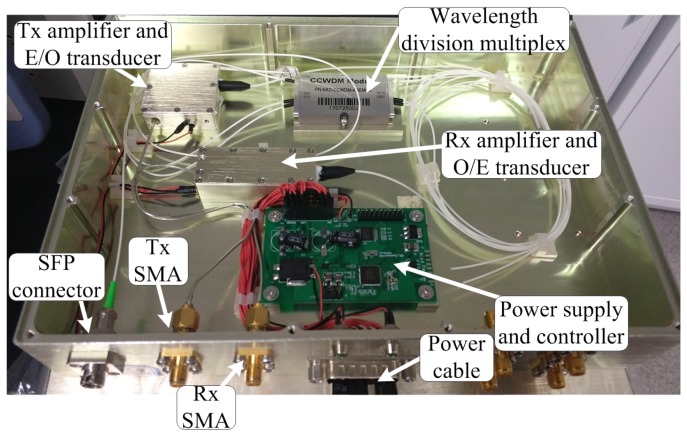
Eagle view of one electronic-optical transducer module.

Figure 8 is an eagle view of the physical realization of the electronic-optical transducer modules. Each module contains three signal IO ports, i.e., a transmitter (Tx) SMA port, a receiver (Rx) SMA port, and an optical SFP port. The Rx port receives the electronic signal and sends it to the electronic-to-optical transducer, where this electronic signal is transformed to modulated light, and fed to the optical fiber connecting the SFP port. On the contrary, the feedback optical signal is received at the same SFP port, then transformed back to electronic signal and sent out at the Tx port.

**Figure 9:**
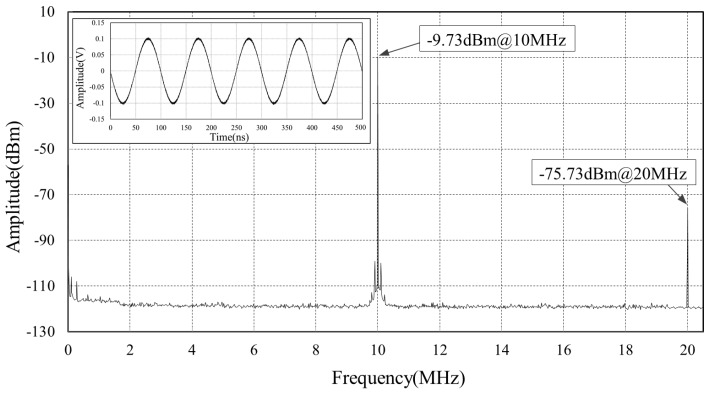
The spectrum of the reference signal before transmitted by an E/O module.

**Figure 10:**
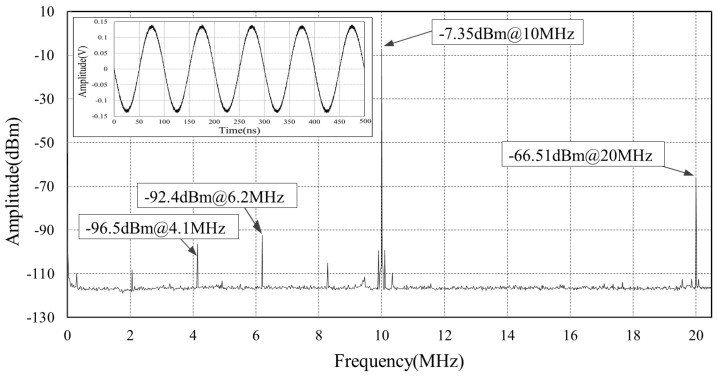
The spectrum of the reference signal after transmitted by 100 m fibers and recovered by another E/O module.

Figure 9 and Figure 10 show the effect of the E/O transducer module on the signal. We use a 10-MHz reference signal feeding to the E/O transducer module. Through a 100 m long fiber, this optical microwave is then received by another E/O module and recovered to electronic signal. Figure 9 depicts the signal before transmitting. The signal power is −9.73 dBm with −75.73 dBm second harmonic spur. Figure 10 gives the spectrum of the recovered electronic signal revealing several spurs around 2 MHz, 4 MHz, 6 MHz, which is introduced by the E/O modules. These low frequency spurs can be filtered by onboard band pass filters and hence neglected. It is worth to note that the recovered signal power at 10 MHz is 2.38 dB larger than that before transmitting, because amplifiers are adopted at both ends of the fiber to recover signals.

**Figure 11:**
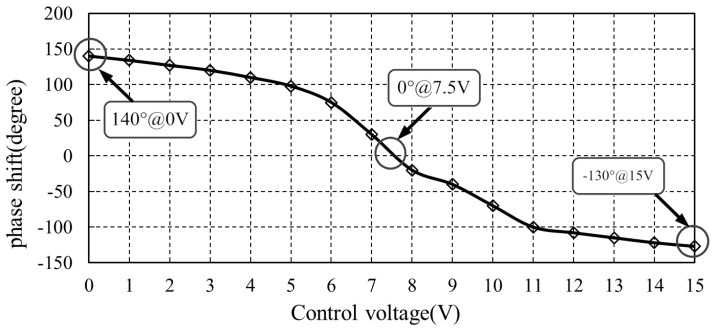
The measured phase shift value versus tunning voltage. For a single phase shifter, the full phase shift range is only from −130° to 140°.

We measure the tunning voltage and phase shift value of one phase shifter and illustrate them in Figure 11. Figure 11 shows that the phase shift monotonically decreases with tunning voltage. When the tunning voltage is set between 7.5 V and 15 V, the phase shift is negative and ranges from 0° to −130°. When tunning voltage is set between 0 V and 7.5 V, the phase shift is positive and ranges from 140° to 0°. That is to say the full phase shift range of a single phase shifter is from −130° to 140°. To guarantee total phase shift range of −360° to 360°, we place two phase shifters at the forward lane and two phase shifters at the feedback lane, achieving a total phase shifter range from −520° to 560°.

**Figure 12:**
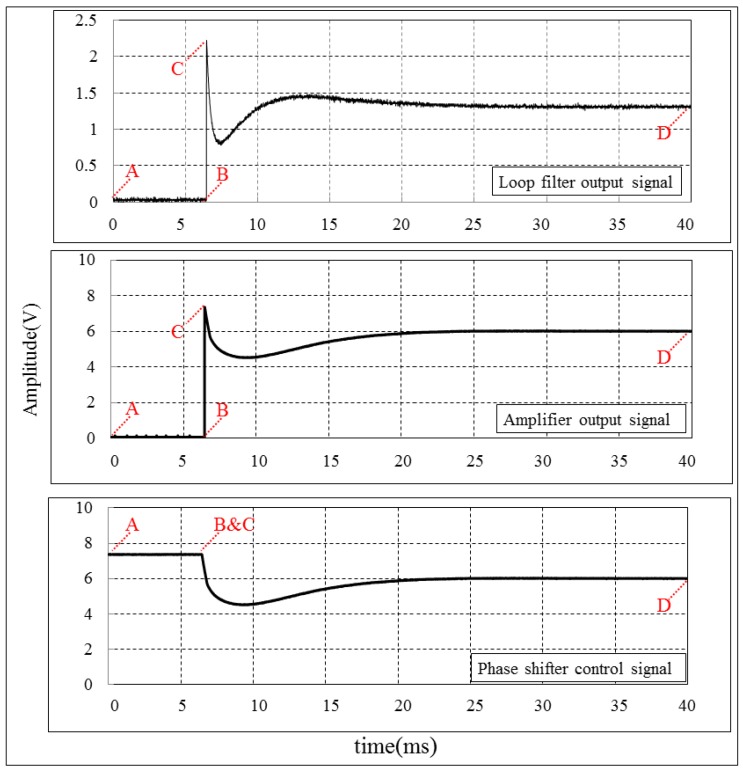
The transient responses of the LPF’s output signal, $v_{cp}$ amplifier’s output signal, and phase shifters’ control inputs. The LPF’s output and $v_{cp}$ amplifier’s output suffer from a rapid voltage change at 6.5 ms, which is caused by the switching.

The transient responses of the LPF’s output, $v_{cp}$ amplifying circuit’s output and phase shifters’ control inputs are shown in Figure 12. During the initial state (curve A–B), the phase shifters’ control inputs are connected to 7.5 V DC voltage. Meanwhile the forward signal and feedback signal are phase misaligned. Hence, the LPF’s output and $v_{cp}$ amplifier’s output are both at 0 V. At point B, the SP2T switch changes the connection of the phase shifters’ control pins from 7.5 V DC voltage to the $v_{cp}$ amplifier’s output and the PLL starts to take effect. At this moment, the $v_{cp}$ amplifier’s output voltage is clamped to 7.5 V (curve B–C). The voltage at the phase shifters’ control pins declines slowly from 7.5 V. Then, the phase shifters simultaneously adjust the phase relation between the forward signal and the feedback signal until they are phase aligned (curve C–D).

**Figure 13:**
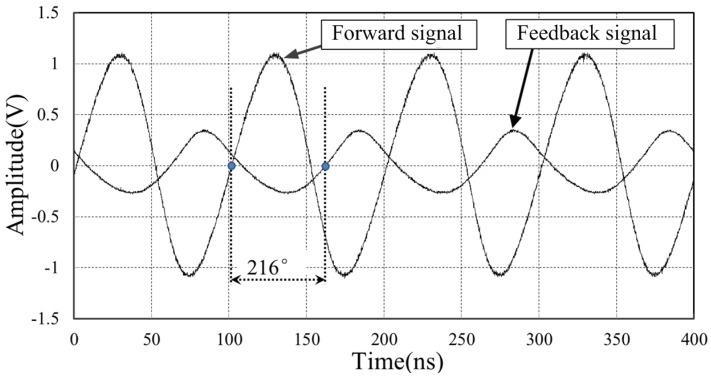
The initial state of the forward signal and feedback signal, where the phase difference is 216°.

**Figure 14:**
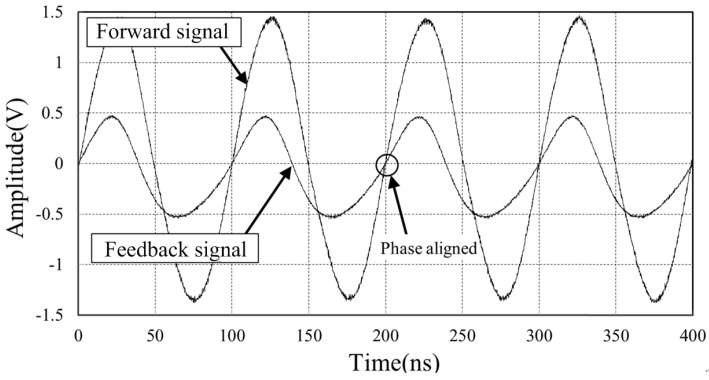
The feedback signal is phase aligned to the forward signal, with the help of phase stabilizer. The residual phase difference is less than 0.4° at 10 MHz.

Figure 13 shows the power-up initial state of the forward reference signal and feedback signal at the central station board. The frequency of reference clock is 10 MHz. The initial phase difference is 216°. Owing to the proposed phase stabilizer, the feedback signal is phase aligned to the forward reference signal as shown in Figure 14. The residual phase difference is less than 0.4°.

**Figure 15:**
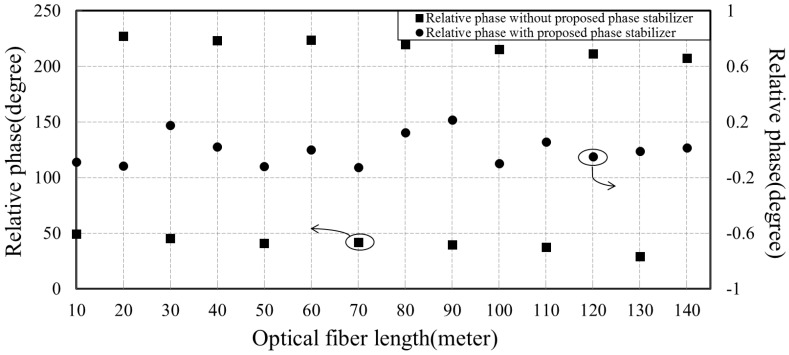
Relative phase difference caused by the fiber length variation. Without phase stabilizer, the phase difference is scattered randomly between 0° and 360°. Using the proposed phase stabilizer, the phase difference is reduced to less than 0.4° at 10 MHz.

The residual phase difference before and after utilizing the proposed phase stabilizer is demonstrated in Figure 15. The wrapped phase difference between the recovered reference signal at the sensor node board and the reference signal at central station board is measured. The sensor node is connected to the central station by optic fibers of different length. The frequency of disseminated reference clock is 10 MHz. We can see that without the proposed phase stabilizer, the wrapped phase difference changes dramatically with the fiber length variation, about 17.14°/m. However, by using the proposed phase stabilizer, the wrapped phase difference is reduced to less than 0.4°.

**Figure 16:**
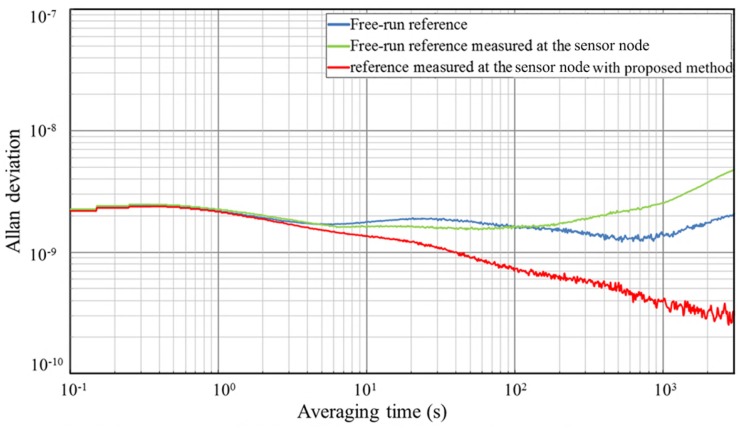
Measured stability of the reference dissemination system. The measured fractional frequency stability (blue line) is the result of the free-run reference signal. The green line shows the result of this reference signal at the sensor node board without phase stabilizer. The red line shows the result with the phase stabilizer.

Furthermore, we set up an Allan Deviation measurement test bench to quantify the stability of recovered reference signal at the remote sensor node board. Figure 16 shows the measured frequency stability of the recovered reference signal. The blue line is the Allan Deviation of the free-run reference signal at the central station board. It has a stability of $2.21\times 10^{-9}$/s, $1.71\times 10^{-9}$/min, and $2.01\times 10^{-9}$/h. This reference is then disseminated to the remote sensor node through fiber links. Without proposed phase stabilizer, the measured Allan Deviation of the recovered reference at the sensor node is shown as the green line, with a stability of $2.26\times 10^{-9}$/s, $1.56\times 10^{-9}$/min, and $4.93\times 10^{-9}$/h. The green curve has similar values to blue curve when the averaging time is less than 100 s. The dominant noise with short averaging time is the white noise and flicker noise introduced by the reference signal source. When averaging time increases, the green curve grows faster than the blue curve. This is caused by the random walk noise introduced by the reference dissemination system and fiber links [31]. The red line shows the effect of the phase stabilizer with a stability of $2.19\times 10^{-9}$/s, $8.58\times 10^{-10}$/min, and $3.42\times 10^{-10}$/h, in which short-time white noise, flicker noise and long-time random walk noise are effectively refrained by the proposed phase stabilizer.

The impact of reference clock phase misalignment on the distributed sonar system performance has been analyzed. To simplify, the simulated sonar system contains only one transmitter array and one receiver array. These two arrays are separated 200 km away from each other. The central station is located with the receiver array. We use geometric dilution of precision (GDOP) as a criterion to verify the performance of our proposed phase stabilizer. GDOP describes the location accuracy of the sonar at every point in the working area. Through the calculation of GDOP, the performance of different subsets in locating target can be measured. A smaller GDOP indicates a better positioning accuracy [32,33,34]. GDOP can be calculated as

(8) $$GDOP=\sqrt{\sigma_{x}^{2}+\sigma_{y}^{2}+\sigma_{z}^{2}}$$

$\sigma_{x}^{2}$, $\sigma_{x}^{2}$, $\sigma_{x}^{2}$ respectively express the standard deviation of target’s positioning error in *x*, *y*, *z* directions and are mainly determined by factors, such as the location errors of the transmitter array and receiver array, elevator angle error, azimuth angle error, and time synchronization error among arrays.

The origin of the Cartesian coordinate is located at the middle of the transmitter array and receiver array. The *z* axis represents the height from seafloor. The simulated sonar system is deployed on the seafloor and the target is 1 km higher than the sonar system. Location coordinates of the transmitter array and receiver array are (−100,000, 0, 0) and (100,000, 0, 0), respectively. The standard deviation of location errors of the transmitter array and receiver array are both assumed 10 m. The standard deviations of elevation angle error and azimuth angle error are both assumed 0.1°.

**Figure 17:**
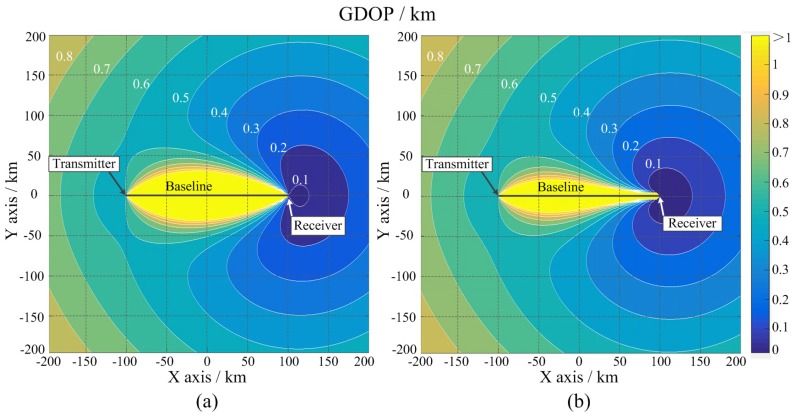
Simulated GDOP of the sonar system with one transmitter array and one receiver array. (**a**) The GDOP with time synchronization error of 50 ms. (**b**) The GDOP of the sonar system with the proposed phase stabilizer.

In the first simulation, the system synchronizes the transmitter array and receiver array initially, but lacking real-time synchronization. The long-term time synchronization error is set to 50 ms. The simulated GDOP is shown in Figure 17a. In the second simulation, we introduce the proposed phase stabilizer and obtain long-term synchronization error of 0.4° for the 10 MHz reference clock, which is equal to 0.11 ns. The simulated GDOP is shown in Figure 17b.

**Figure 18:**
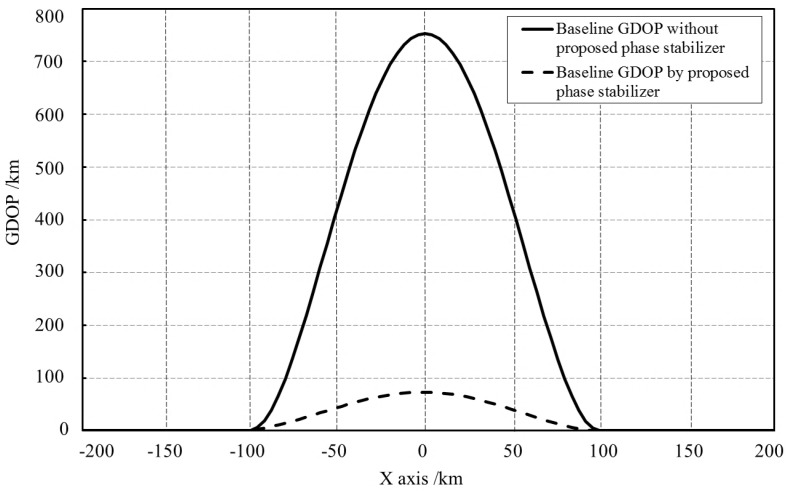
The GDOP value along the baseline.

Figure 17 reveals that the GDOP is distributed symmetrically versus the *x* axis (baseline). The GDOP distribution can be divided into three zones: Baseline zone, where the GDOP value is pretty high. The GDOP value increases rapidly when the target approaches the baseline. The GDOP value on the baseline is too high to detect target; High accuracy zone. The GDOP value around the receiver array is very low. The GDOP decreases rapidly when the target approaches the receiver array and the system can detect objects with high accuracy. The outer zone is far away from the baseline and receiver array. In outer zone, the GDOP value is moderate and the contour lines are like circles with the center of the circles located near the receiver array. The GDOP value increases gradually as the target leaves the receiver array. Comparison between Figure 17a,b reveals that, with the proposed phase stabilizer, the baseline zone shrinks obviously and the high accuracy zone extends, meaning that the overall detecting accuracy is improved. The GDOP value along the baseline is also drawn in Figure 18. Without the proposed stabilizer, the GDOP’s peak value is 750 km. By using the phase stabilizer, the peak value is decreased to 80 km.

## 4. Conclusions

We have described an electronic method to stabilize the phases of reference signals at different sensor nodes for distributed underwater sonar systems. Our method can effectively compensate the phase differences caused by fiber length variations. The experimental results have validated that our method can realize phase synchronization with only 0.4° remaining error for a 10-MHz reference clock, which is acceptable for most distributed sonar applications. We also demonstrate an Allan Deviation test bench to quantify the impact of the proposed method on noise and show that our method can effectively stabilize the signal with short-time white noise and flicker noise and long-time random walk noise. At the end of this paper, we also analyze the impact of the reference clock’s phase error on the system range detection accuracy, which indicates that the proposed phase stabilizer can greatly improve detection accuracy of sonar systems.
